# Meetings of Societies

**Published:** 1901-12

**Authors:** 


### MEETINGS OF SOCIETIES.

### Bristol Medico-Chirurgical Society.

ANNUAL MEETING, *October 9th, 1901.*

After a vote of thanks to the retiring President (Dr. D. S. Davies) had been unanimously passed, Dr. BARCLAY J. BARON took the chair and gave the introductory address (see p. 289), for which a cordial vote of thanks was afterwards given.

The HONORARY SECRETARY (Mr. J. Paul Bush) read his Annual Report. He had to record that the Society had lost three of its members—all Past-Presidents of the Society—through death; viz., Drs. W. J. Fyffe, S. H. Swayne, and A. E. Aust Lawrence. The balance in hand was £63 7s. 9d., together with £75 1s. 7d. on the *Journal* account. The report was adopted, and thanks given to the Secretary for his services.

The Editorial Secretary (Mr. James Taylor), in his report for the year, stated that the cost of the production of the *Journal* had slightly increased owing to the number of illustrations published.

The Honorary Librarian of the Medical Library (Mr. L. M. Griffiths) gave his annual report, and stated that the Society's Library contained 9,371 volumes, and the Bristol Medical Library, of which the former was a part, contained 20,390 volumes and 227 periodicals.

The following officers were chosen for the ensuing year: President-Elect, Mr. G. Munro Smith; Honorary Secretary, Mr. J. Paul Bush; Honorary Librarian, Mr. L. M. Griffiths; Members of Committee, Dr. J. Michell Clarke, Mr. J. Dacre, Dr. B. M. H. Rogers, Mr. J. Taylor, Dr. H. Waldo, and Dr. P. Watson Williams; Members of the Library Committee, Mr. L. M. Griffiths, Mr. G. Munro Smith, and Mr. J. Taylor.

---

*November 13th, 1901.*

Dr. Barclay J. Baron, President, in the Chair.

Mr. T. Carwardine showed two patients: one, a case of sarcoma with spontaneous recovery; the other, a case of multiple lipomata. The first patient was a boy, 16 years of age. A tumour having the naked-eye characters of a sarcoma had been removed from the inner side of the right arm and from the axillary region adjoining. The removal was not complete, infiltrated muscle being left behind. The existence of enlarged glands in the left posterior triangle and left axilla had contra-indicated a "fore-quarter" amputation. It was a year since the tumour had been removed. The scar was healthy and the enlargement of the glands had disappeared. A section was shown under the microscope, and the appearance was that of a myxo-sarcoma. The patient had taken arsenic since the operation. The man with multiple lipomata had made the interesting statement that on two occasions the tumours had disappeared.—Mr. C. A. Morton considered that the evidence of the glands on the left side being sarcomatous was not conclusive.—Dr. Lacy Firth mentioned a case of primary sarcoma of the sartorius muscle he had treated by removing the upper half of the affected muscle. The patient, an old woman, developed erysipelas after the operation; but in spite of that and the toxins it must have formed, the tumour very quickly recurred. —Mr. Roger Williams thought there was a tendency in the profession for men to believe what they wished to believe. He had never found in his researches that a case of malignant tumour had spontaneously disappeared, and he did not believe that such a disappearance ever occurred. It was the difficulty in diagnosis which led to reports of such disappearances. He thought there was no satisfactory evidence of the present case being one of sarcoma. Also, there were still nodules near the scar in the case, and nodular glands were present, and these might be due to recurrence. In the case of the man with fatty growth the analogies were with obesity and myxœdema, and the swellings were not true tumours. Probably they were the result of a tropho-neurosis. The speaker showed photographs of some similar cases he had seen. The leg seemed always to escape being involved. Such fatty growth was physiological in the Hottentots and some negroes.—Mr. Carwardine, replying, said that the appearance of the growth on section was that of a malignant tumour, it infiltrated muscle, and the microscope supported that view.

Dr. E. C. WILLIAMS showed a child with an interesting cardiac murmur. The patient was 8 years old. There was no history of rheumatic fever or scarlet fever. The heart was only slightly enlarged, and that in an upward direction. The murmur was a loud one, continuous through the cardiac cycle, and heard best at the base, and with maximum intensity over the third left costal cartilage. There was a slight thrill in the third left intercostal space. The only symptom was some shortness of breath on exertion. There was no cyanosis. He put the question whether the case was one of pulmonary stenosis or of patency of the ductus arteriosus.—Drs. Michell Clarke, Stack, Kenneth Wills, T. Fisher, Markham Skerritt, and Elliott discussed the case.

Dr. W. H. NEWNHAM showed the following specimens:—(1) The parts removed in a case of ruptured tubal pregnancy. The patient had suddenly passed, a few days previously, into a state of shock and pulselessness. She rallied before coming to the Hospital. Nothing was felt on abdominal examination. At the operation blood welled up through the wound very copiously. Abdominal irrigation had been necessary to remove the clots. The patient was now well. (2) A pregnant fibroid uterus he had removed from a woman who was in the fifth month of pregnancy. The cervix was very high up, and the pelvis blocked by the tumour. The patient did well after the operation. (3) A uterus presenting several fibromata.—Dr. JAMES SWAIN thought washing out the abdomen with saline fluid a valuable step in operation for ruptured tubal pregnancy. Leaving fluid in the abdomen combatted shock. He had had a case of pregnancy complicated with fibroid disease in which the diagnosis was difficult, and which, like that related above, had been considered to be a case of extra-uterine pregnancy.—Mr. T. CARWARDINE showed a fœtus he had recently removed from the abdomen in a case of ruptured tubal pregnancy.—Dr. NEWNHAM replied that he preferred not introducing fluid into the abdomen if it could be avoided. He had diagnosed the fibroid disease in the case related, and the fœtal heart had been heard before operation, showing that the patient was pregnant.

Dr. T. FISHER showed two specimens—the first of actinomycosis of the liver and lungs, obtained from a case of appendicitis which had originated from perforation of the appendix by a pin. The pin had been found when the abscess had been opened during life. The subsequent course of the case was like that of pyæmia. The scattered lung abscesses were actinomycotic. The second specimen was a portion of the liver of a man who had died of emphysematous gangrene, showing cavitation from numerous small collections of gas in the organ.—Dr. JAMES SWAIN gave further details of the case of actinomycosis. He had operated several times. The pin had been found at the first operation in an abscess, but the operation had not shown where it came from. The boy went from bad to worse, developed pyæmic symptoms, and died in three or four months. The pus had shown only the bacilli communis and pyocyaneus.—Dr. MARKHAM SKERRITT related the case of a boy who for a long period had had an elevated temperature, for a long time unexplainable. Then slight enlargement of the liver was developed, and the diagnosis of actinomycosis suggested. The liver, where palpable, had a doughy feel. The case proved to be one of that affection.—Mr. PAUL BUSH mentioned a case he had repeatedly operated upon for actinomycosis during the past few years. The man still came up from time to time.

—Dr. FISHER stated that a perforation had been found in the appendix at the *post mortem*, which had probably been caused by the pin.

Dr. WALTER C. SWAYNE read a paper on three cases of abdominal section. Case I.: In this woman a few weeks after parturition the abdomen was opened for a large tumour, the size of a five-months' pregnant uterus. The tumour was found to have a peritoneal covering, and ultimately, after tapping, proved to be a large hydro-nephrotic kidney. It was removed by the abdominal route. The specimen was shown. A good recovery was made. Case II.: In this case the abdomen was opened soon after parturition. The patient was suffering from increasing abdominal pain, the temperature was rising, and a ruptured or twisted ovarian cyst was suspected. Laporotomy revealed a sausage-shaped swelling in the right broad ligament, which appeared to have resulted from the rupture of a blood-vessel in the ligament. Complete cessation of pain followed the operation, and the patient recovered well, except for an attack of phlegmasia fourteen days later. Case III.: The patient was a chronic invalid with irreducible retroversion of the uterus. Hysteropexy was advised. Both ovaries and tubes were buried in adhesions and the former cystic. They were removed and the uterus fixed in good position. On the third day the gauze drain, which had been applied according to the method of Mickulicz—a layer of gauze like a bag being first applied and then stuffed with gauze strips,—had to be removed under an anæsthetic.—Mr. C. A. MORTON alluded to the superiority of the method of emptying and removing hydro-nephrotic swellings by the lumbar route when possible.

Mr. JAMES TAYLOR read a paper on X-rays in the diagnosis of renal calculus. At the Bristol Royal Infirmary, during the last twelve months, he had been able to show the presence of renal stone in five cases. It was usually easy, with two to three minutes' exposure, to show such stones. He considered that a negative result was of some significance. The value of the method for detecting stones was great, in enabling surgeons to avoid the oversight of a second or third stone after one had been removed; and one of the cases recorded particularly illustrated this advantage of the use of the X-rays. Notes of the cases were read and radiographs of each shown. One case was in a boy only 13 years of age.—Mr. PAUL BUSH emphasized the importance of this mode of detecting the presence of more than one calculus.—Dr. JAMES SWAIN was not convinced of the negative value of these photographs. He alluded to the fact that phosphatic calculi yielded a deeper shadow than uric acid calculi, though the phosphatic varieties were less dense than those of uric acid. This was an exception to the usual rule, that X-rays gave a deeper shadow in proportion to the density of the objects examined. It was probably due to a special opacity of the lime salts in the phosphatic calculi.—Mr. C. A. MORTON mentioned a case in which he had found a stone in a stout man after failure of the X-rays to demonstrate its presence.—Dr. EDGEWORTH alluded to the case of the boy with stone which had been mentioned, and said that the hæmaturia and the other clinical symptoms had not indicated even which kidney was the source of the trouble, yet the X-rays had done this easily.—Dr. FISHER alluded to the presence of uric acid crystals in the urine of children, and to the probability of this causing hæmaturia.—Mr. FLEMMING alluded to the transparency to X-rays of plaster-of-Paris, *e.g.* fractures were visible through splints of this material, and asked what was the cause of the opacity of phosphatic calculi.

J. LACY FIRTH.

